# Preparing for and Not Waiting for Surgery

**DOI:** 10.3390/curroncol31020046

**Published:** 2024-01-23

**Authors:** Andrew Bates, Malcolm A. West, Sandy Jack, Michael P. W. Grocott

**Affiliations:** 1 Perioperative and Critical Care Medicine Theme, NIHR Southampton Biomedical Research Centre, University Hospital Southampton/University of Southampton, Southampton SO16 6YD, UK; a.bates@soton.ac.uk (A.B.); m.west@soton.ac.uk (M.A.W.);; 2 Faculty of Medicine, University of Southampton, Southampton SO16 6YD, UK

**Keywords:** cancer surgery, prehabilitation, perioperative medicine, functional capacity, physical fitness, exercise, nutrition, psychology, cognitive

## Abstract

Cancer surgery is an essential treatment strategy but can disrupt patients’ physical and psychological health. With worldwide demand for surgery expected to increase, this review aims to raise awareness of this global public health concern, present a stepwise framework for preoperative risk evaluation, and propose the adoption of personalised prehabilitation to mitigate risk. Perioperative medicine is a growing speciality that aims to improve clinical outcome by preparing patients for the stress associated with surgery. Preparation should begin at contemplation of surgery, with universal screening for established risk factors, physical fitness, nutritional status, psychological health, and, where applicable, frailty and cognitive function. Patients at risk should undergo a formal assessment with a qualified healthcare professional which informs meaningful shared decision-making discussion and personalised prehabilitation prescription incorporating, where indicated, exercise, nutrition, psychological support, ‘surgery schools’, and referral to existing local services. The foundational principles of prehabilitation can be adapted to local context, culture, and population. Clinical services should be co-designed with all stakeholders, including patient representatives, and require careful mapping of patient pathways and use of multi-disciplinary professional input. Future research should optimise prehabilitation interventions, adopting standardised outcome measures and robust health economic evaluation.

## 1. Introduction

Cancer treatment heavily relies on surgery, encompassing preventative, diagnostic, curative, palliative, and reconstructive interventions [1]. However, these procedures, while essential, introduce considerable trauma and physiological disruption, posing substantial risks to patients’ physical and psychological well-being. Despite advancements such as enhanced recovery programs, minimally invasive surgical techniques, and robotic surgery, elective cancer surgery remains associated with a notable mortality risk [2]. The prevalent focus on traditional short-term reporting potentially obscures the full scale of the issue, given the consideration of ‘late mortality’ occurring between days 31 and 90, or even later [3]. Estimates of postoperative morbidity vary based on factors such as heterogenous outcome reporting, level of hospital infrastructure [4], surgical site, and complexity [5]; however, the considerable impacts on patients, families, and global healthcare systems are widely acknowledged [2,5,6]. Urgent and elective cancer surgeries exhibit similarly unfavourable outcomes [7]. Notably, over half of patients aged 60 and above who undergo major abdominal surgery fail to regain their preoperative functional capacity, quality of life, or physical fitness [8,9]. Perioperative risk is multi-factorial, a function of preoperative condition of the patient, surgical complexity, and anaesthetic administration. Cancer patients face particular burden due to the deconditioning nature of disease, and neoadjuvant treatment and potential for multiple exposures to anaesthetics during diagnostic and treatment phases [10]. The projected economic toll from 2015 to 2030, solely due to productivity loss, is estimated at USD 12.3 trillion, amplifying health inequalities and spiralling economic harm [4].

Global population estimates anticipate a doubling of individuals aged over 65 between 2025 and 2050, reaching 1.6 billion [11]. As a consequence of the striking association between old age and cancer incidence, the projected annual count of cancer surgeries worldwide is expected to rise from 30 million in 2015 to 45 million by 2030 [1].

A patient’s preparedness for surgery’s physiological and psychological impact is not guaranteed. Historically, healthcare systems have prioritized the operation and disease itself. While historically healthcare prioritised the operation and disease, a compelling case supports postoperative outcomes being primarily influenced by patient resilience, i.e., their ability to counteract perioperative stressors [12]. This paradigm shift positions the surgical response as the primary ‘disease process,’ urging a recalibration of perioperative care to centre on optimising patient resilience at the time of contemplating surgery. Perioperative medicine now encompasses comprehensive support from initial suspicion of diagnosis to full recovery [13]. The interval between diagnosis and surgery presents an opportunity to tailor care for the changing patient demographic with intricate health requirements. Achieving this entails meticulous comorbidity management, arranging suitable enhanced care facilities, supporting health-enriching behaviours, and fostering informed discussions regarding the appropriateness of surgery, particularly when potential harm might outweigh the benefits. Regrettably, the prevalent care models rarely align with these risk-focused goals, often prioritizing siloed health system concerns such as treatment timeline, clinic availability, operating room capacity, and postoperative care resources. Reconfiguring surgical processes to facilitate patient-centric pathways, rooted in comprehensive risk assessment, can yield manifold advantages [14]. At this critical juncture, facing escalating cancer care demands and limited resources, adopting a business process re-engineering approach to perioperative medicine aligns with the widely-adopted “Quintuple Aim” of healthcare, i.e., enhancing care experiences, bolstering population health, reducing per capita healthcare, addressing clinician burnout, and advancing health equity [15,16,17].

This review has multiple objectives, specifically, raising awareness of the global public health concern, proposing a systematic framework for patient phenotyping and perioperative risk evaluation, and emphasizing the potential of personalized prehabilitation plans to mitigate risk based on international expert consensus guidelines. The manuscript, structured into two parts, initially focuses on established patient-level risk factors, broadly categorized under “functional capacity,” discussing their implications for perioperative outcomes and providing a concise overview of screening and assessment procedures. The subsequent section explores how prehabilitation can act as a risk-mitigating strategy, complementing interventions like managing comorbidities and facilitating smoking and alcohol cessation. It is crucial to note that while presented in a perioperative context, these actions hold promise for broader health benefits through longer-term behaviour change, making the perioperative period an ideal teachable moment for clinicians to positively impact multiple health domains.

## 2. Functional Capacity: Navigating Definitional Controversies of Perioperative Risk Assessment

Traditional preoperative risk assessment, focused on surgical complexity, coexisting medical conditions, and more recently incorporating an evaluation of functional capacity, often relies on subjective judgments of physical fitness [18]. However, resuming daily activities after surgery necessitates an integrated physiological response, involving cardiopulmonary, neuromuscular, musculoskeletal, metabolic, and psychological systems. Contemporary evidence advocates for a broader risk definition, extending to proficiencies essential for meaningful postoperative function, including physical fitness, nutritional status, psychological well-being, cognitive function, and frailty [19,20,21]. Poor dietary intake, nutritional status and sedentarism can induce inflammatory, metabolic, and endocrine processes that promote cancer development through accumulated DNA damage, diminished cancer apoptosis, and sustained proliferative signalling [22,23]. A sensitive screening process should identify at-risk individuals for comprehensive assessment by a proficient clinician within a healthcare facility. Given evolving evidence, achieving accurate and scalable risk assessment requires a comprehensive reorganization of perioperative medicine services [14].

In perioperative medicine, the concept of functional capacity is evolving from a single-dimensional to a multi-faceted evaluation, requiring further research on measurement methodologies, component weighting, and feasibility for widespread implementation. Balancing comprehensiveness, practicality, and sensitivity to local context is crucial in defining and utilizing functional capacity assessments for improved perioperative care. In this review, we present established patient-level risk factors and propose a stepwise risk identification framework to guide outcome-determinative interventions.

### 2.1. Physical Fitness

Empirical evidence from the 1990s linked objectively measured low physical fitness with heightened postoperative risk in elderly patients undergoing major intra-cavity surgery [24]. Recent systematic reviews and meta-analyses have affirmed these findings across diverse cancer surgical populations and procedures [25,26,27]. The well-established link between sedentarism and cancer development further promotes the imperative to adequately establish preoperative physical fitness. Moreover, neoadjuvant treatment (NAT) is integral to preoperative cancer care, aiming to enhance circumferential margins. However, NAT introduces cardiovascular deconditioning through combined effects of direct cardiotoxicity, unmasking of compensatory mechanisms of cardiac dysfunction, and mitochondrial degradation [25,28,29].

In the perioperative domain, physical fitness assessment often relies on subjective clinician estimates using the American Society of Anesthesiologists (ASA)’s Physical Status Classification System or metabolic equivalent of tasks (METs), determining fitness to proceed if the patient exceeds four METs without symptoms [30]. Moreover, there is significant discordance between clinician-assessed and patient-reported exercise capacity [31]. Such techniques inaccurately gauge patient fitness, limiting predictive utility [27,32], and have prompted calls for systematic and objective screening at contemplation for surgery [12,13].

Screening: Performing a gold standard assessment of physical fitness on all patients awaiting surgery is likely to be hampered by resource limitations and may not be necessary. An incremental approach involving universal screening to identify individuals needing comprehensive assessment is recommended. Initiating screening close to diagnosis, utilising concise digital tools for scalability, is advisable. The screening instrument should have sufficient sensitivity to identify high-risk patients, facilitating referral for specialised assessment and personalized care direction [33]. Various self-reported screening tools, extensively utilized and validated within surgical populations, are compiled in Table 1. While there is controversy around the accuracy of such tools, aggregate data from PROMs have been incorporated into routine healthcare practices and perioperative research for an extended period. Notably, in the United Kingdom, these data have been used to evaluate the performance of healthcare providers within the primary care Quality and Outcomes Framework (QOF) [34], and in surgical populations in the Patient Reported Outcome Measures (PROMs) initiative [35].

Assessment: Self-reported screening should complement, not substitute for, objective physiological evaluation [37]. Individualised assessment demands a comprehensive scrutiny of physical fitness using validated clinical measurement techniques, administered by registered healthcare professionals, best directed at patients surpassing prognostically significant screening thresholds.

Cardiopulmonary exercise testing (CPET) emerges as the established gold-standard for preoperative risk evaluation, encompassing a dynamic integrated appraisal of cardiopulmonary, neuromuscular, metabolic, and musculoskeletal systems [38]. Impaired CPET performance predicts immediate postoperative complications, enduring morbidity, and mortality risks, while also uncovering undiagnosed pathologies and providing parameters guiding prehabilitation programs. Key focus areas include oxygen uptake at the anaerobic threshold and ventilatory capacity, displaying the best predictive potential [25,38].

In instances where the logistical demands, expenses, or expertise required for preoperative CPET hinder its implementation, results from alternative assessments like the six-minute walk test (6-MWT) [39] and Incremental Shuttle Walk Test (ISWT) [40] correlate with CPET-derived results, and association with postoperative outcomes [25]. While natriuretic peptide, a biomarker of cardiac dysfunction, displays a modest correlation with CPET variables [27], its application in preoperative testing shows potential in predicting postoperative cardiac complications, particularly for patients with concurrent cardiac comorbidities [41].

### 2.2. Nutritional Status

Malnutrition, an imbalance between nutrient/energy intake and requirements, leads to reduced metabolic reserve, sarcopenia, cachexia, and compromised physical fitness [42,43]. Strong associations exist between weight loss, low muscle mass, and reduced survival in various cancers, with patients experiencing these issues surviving about 8 months compared to 28 months for those without [44,45]. Cancer cachexia, affecting 50–80% of cancer patients, arises from tumour-induced anorexia, catabolic effects, altered nutrient metabolism, gastrointestinal tract obstruction, and reduced food intake [46]. Inadequate dietary intake due to pain, anxiety, and depression deserves attention.

The latest guidelines from the European Society for Clinical Nutrition and Metabolism (ESPEN) underscore the link between nutritional status and postoperative outcomes, with malnourished surgical patients experiencing elevated morbidity, mortality, prolonged hospital stay, unplanned readmission rates, and increased inpatient care costs [47,48]. Loss of skeletal muscle mass and function (sarcopenia) is associated with reduced overall survival and increased risk of postoperative complications, across a range of cancer types [44,49]. Patients facing gastrointestinal and head and neck surgery face the highest risk of malnourishment due to the cancer process, effects of systemic anti-cancer treatment and malabsorptive states [49,50]. Severe malnutrition was found in 33% of patients undergoing elective gastric or colorectal cancer surgery, with a positive association with 30-day mortality [50], while preoperative nutritional risk doubles the chance of 30-day readmission [51]. Addressing malnutrition-mediated surgical risk before surgery, even if causing a brief delay, supports a proactive shift to nutritional screening, targeted assessment, and personalized intervention.

Screening: Acknowledging the documented link between nutritional status and postoperative outcomes, the ESPEN recommends universal preoperative screening and targeted assessment [47]. Nutritional screening, a scalable preliminary step, aims to identify individuals at malnutrition risk or with specific nutritional needs. A systematic literature review identified 32 nutritional screening tools, with no consensus on the ideal preoperative tool for “at-risk” surgical patients [52,53]. Clinicians should select a tool that is suitable for their context, quick, easily interpreted, including components related to nutritional condition, stability, potential deterioration, and likely deficits due to disease progression [54]. BMI-adjusted weight loss grading and the Patient-Generated Subjective Global Assessment (PG-SGA) are common, with the latter’s short form used for screening, incorporating self-reported weight loss, dietary intake, symptoms, and function [55,56]. However, caution should be applied as unchanged BMI can mask concurrent sarcopenia and increase in body fat, classified as sarcopenic obesity, an independent predictor of poor postoperative outcome [57]. Those at risk should progress to additional clinician assessment components. A representative sample of candidate measures is listed in Table 2.

Assessment: After identifying an at-risk individual through screening, a comprehensive nutritional assessment is necessary. Conducted by trained healthcare professionals, this assessment involves a detailed examination of nutrition impact symptoms, functional status, disease burden, metabolic impact, and a physical examination to maximize reliability [62]. Various approaches can be used in isolation or combined, based on clinical judgement; clinical history taking, functional assessment, anthropometrics, body composition, biochemical evaluation, and validated nutritional indices [63]. The Subjective Global Assessment (SGA) [59] categorises patients as well-nourished, mildly/moderately malnourished, or severely malnourished, by considering nutrient intake, weight loss, symptoms affecting intake or absorption, functional capacity, metabolic requirement, body composition, oedema, and ascites.

Non-invasive techniques like Bioelectrical Impedance Analysis (BIA) and Dual-energy X-ray absorptiometry (DEXA) estimate body composition (primarily measuring lean muscle mass) associated with postoperative outcome [64,65]. Computerized tomography and magnetic resonance imaging, though costly and requiring skilled interpretation, determine skeletal muscle index, particularly at the third lumbar vertebrae [66]. Imaging techniques, especially when combined with functional strength assessments like handgrip, strongly predict postoperative complications [66]. Routine preoperative blood tests, including electrolytes, blood urea nitrogen, glucose, lipid, and visceral protein profiles, can determine nutritional status with skilled interpretation [67]. Malnutrition adversely impacts postoperative outcomes, necessitating skilled assessment for timely intervention, as covered in the prehabilitation section below.

### 2.3. Psychological Health

The psychological impact of receiving a cancer diagnosis and facing surgery should not be underestimated [20]. Cancer diagnosis and surgery have a substantial psychological impact, with around 50% experiencing clinically significant distress across various cancer types [68,69]. A meta-analysis reported mood disorders in 30–40% of hospital inpatients, with no significant differences between palliative and non-palliative settings [69]. Pooled data from 16 prospective studies, reporting 4353 cancer related deaths, revealed that higher levels of distress were associated with a 32% greater mortality risk [70]. A 2023 systematic review reported that presence of psychological distress was predictive of reduced survival across 11 of the 13 reviewed studies [71].

Depression and anxiety are associated with poorer outcomes after cancer surgery, including pain [72], delayed wound healing [73], and increased length of hospital stay [74]. High depression and low self-efficacy to self-manage health conditions at diagnosis is predictive of lower quality of life, and can impact treatment option decision-making and reduce mental health for up to two years after surgery [75,76].

A systematic review of 16 studies by Mavros et al. reported heterogenous measures of distress and postoperative outcome; however, there was consistent association between the presence of one or more components of psychological distress and poorer early postoperative outcomes, up to 30-days following surgery [73]. A review of 13 studies by Rosenberger et al. reported that mood, anxiety, and depression predicted short term postoperative outcome, length of hospital stay, self-reported recovery, and long-term pain [77]. Importantly, both of these systematic reviews [73,77] reported association between positive psychological traits and improved recovery. Self-efficacy, low pain expectation, external locus of control, optimism, religious faith, and anger control were associated with favourable postoperative outcomes, suggesting that altering the psychological well-being of people with cancer prior to surgery may have the potential to promote better recovery [20] and improve ongoing compliance with treatment.

Biologically plausible mechanisms involve distress affecting protective systems against cancer progression and wound healing, including the inflammatory response, immunological function, dysregulation of the HPA axis, increased cortisol concentration, and inhibition of DNA repair [78,79]. Moreover, individuals with psychological distress are more likely to adopt risky behaviour such as smoking [80], alcohol use [81], and poor diet [82].

Screening: Recognizing psychological distress as the “6th Vital Sign,” international guidelines emphasize its consideration alongside traditional physiological measures [83,84,85,86]. Integrating psychological care into cancer treatment is crucial, involving evaluation of distress levels and psychosocial needs, followed by appropriate referrals for assessment and treatment. Screening should be fast, simple, and digitalised for scalability [33,87]. However, while there is consensus for the need and a broad framework, there is no agreement on the method used or most appropriate tools [88].

Screening tools used widely in psycho-oncology include the National Comprehensive Cancer Network Distress Thermometer [89], a simple Likert scale with patients asked to rate distress in the last week from 0–10, with a score ≥4/10 triggering referral for more in-depth assessment. Other short-form screening tools, such as the Patient Health Questionnaire-2 [90], Distress Thermometer [91], and Hospital Anxiety and Distress Scale [92] are reliable and acceptable to clinicians and patients [93,94]. These tools have established cut-offs which indicate the need for more detailed assessment and, when indicated, psychological intervention.

Assessment: The international psycho-oncological guidelines broadly recommend a stepwise approach to evaluation of psychological health but lack detailed articulation of clinical pathways [83,84,85,86,95]. A consensus exists for undertaking a more formal assessment of psychological health, with those patients identified at risk following screening. This should be undertaken by a registered psychological professional [33]. Attempts have been made to develop specific psycho-oncological assessment tools [96], but more research is needed to determine validity. Multiple validated tools can be used to support the assessment, such as the Generalized Anxiety Disorder Assessment (GAD-7) [97] and the Patient Health Questionnaire (PHQ-9) [98] for anxiety and depression screening, respectively. The prevalence of symptoms of post-traumatic stress disorder may indicate use of a validated PTSD screening tool. Operational models exist but require rigorous testing in RCTs [95,99]. In addition, professionals should consider substance-induced causes of anxiety.

### 2.4. Frailty

Frailty is a widely used term describing a cumulative, multi-factorial health decline that heightens vulnerability to further deterioration after a stressor event [100,101]. Often viewed as age-related decline, frailty is more accurately a biological expression shaped by genetic and environmental factors, influenced by lifestyle choices like physical activity, diet, smoking, and alcohol use [100]. Among the frail, acute medical events lead to disproportionate changes in health status, with cancer patients particularly susceptible to the cumulative psycho-physiological insults asserted by oncogenesis, anti-cancer treatment, malabsorptive nutritional states, and disease-related symptoms [102]. Characterised by weakness, fatigue, decreased mobility, and cognitive impairment, frail patients have increased vulnerability to falls, delirium, disability, institutionalisation, morbidity, and death [100,103]. Both frailty and cancer incidence rise with age, highlighting the relevance of frailty to cancer surgery services amid a rapidly aging population and an increasing age of people with cancer undergoing surgery [104].

Frailty increases the risk of poor postoperative outcomes, even following minor procedures [21,105,106]. In a study of over 23,000 patients undergoing non-cardiac surgery, frailty was linked to a 1.5-fold increase in postoperative healthcare cost [107]. A meta-analysis of 45,000 frail patients undergoing non-cardiac surgery revealed mortality rates of 1.55% following the lowest-stress surgery (e.g., cystoscopy). The rate of 180-day mortality reached 43% for very frail patients undergoing moderate-stress procedures (e.g., laparoscopic cholecystectomy) [108]. Despite this robust association, frailty is inadequately considered during perioperative assessment and subsequent shared decision-making, risking patients undergoing inappropriate procedures or being excluded from potentially beneficial ones that could be supported by interventions that modify frailty characteristics [109,110,111].

Screening: Clinicians should adopt a stepwise approach by universal screening of cancer patients aged over 65, and younger patients where indicated, as early as possible in the surgical pathway, preferably using electronic systems [19]. Frailty phenotyping involves two approaches: profession-led evaluation, considering physical, psychological, cognitive, and nutritional aspects, and self-reported patient screening tools. Despite various frailty screening tools (Table 3 contains a non-exhaustive summary of validated frailty screening tools) and a universally accepted definition, no gold standard screening tool has been established [36]. Clinicians are urged to shift from age-based judgments, opting for tools that better capture the meaningful concept of frailty [100], considering brevity, sensitivity, and specificity relevant to their population, aims, and clinical context. High-risk patients should undergo early, detailed assessment in a perioperative clinic to facilitate SDM discussions and develop mitigating strategies.

Assessment: The Comprehensive Geriatric Assessment (CGA) is considered the gold standard for frailty assessment, encompassing interdisciplinary evaluation of medical, nutritional, social, psychological, and functional aspects [102,115]. A systematic review found that utilizing CGA improved functional recovery, reduced mortality, and lowered healthcare costs in both elective and emergency surgical services [116]. The United Kingdom Centre for Perioperative Care recommends a CGA for patients with a Clinical Frailty Scale (CFS) score ≥5 [109]. If resource or staffing constraints hinder CGA, perioperative clinicians can enhance the reliability and specificity of a positive frailty screen by including functional assessments like gait speed, sit-to-stand, timed up and go, balance, and grip strength.

### 2.5. Cognitve Function

While cognitive decline is a normal process of aging, the rate and underlying aetiology are highly heterogenous. Severity lies on a continuum with three widely accepted phases. Pre-clinical or Subjective Cognitive Decline (SCD) is characterised by self-reported cognitive decline without measurable symptoms [117,118]. Mild cognitive impairment (MCI) is diagnosed with cognitive changes, abnormal function, and no dementia [119]. MCI sufferers can be sub-grouped as amnestic or non-amnestic MCI, with implications for understanding and completing postoperative recovery plans. Finally, dementia is the most severe stage, with deficits across multiple cognitive and functional domains [120]. Progression is not inevitable, and while SCD and MCI phases may have slow progression, a recent review noted accelerated neurocognitive decline in cancer patients over 65, making perioperative clinicians potential early identifiers [120,121].

Global dementia cases are projected to triple to 130–175 million by 2050, with significant regional variations [122].

Each stage of cognitive impairment increases healthcare utilization, institutionalization, and mortality. Preoperative cognitive impairment predicts delirium, postoperative complications, 12-month mortality, 30-day readmission, discharge to assisted care, and long-term neurocognitive issues [123]. Postoperative cognitive decline accelerates in elderly patients with pre-existing cognitive impairment [124]. A 2018 state of the science summit coined the overarching term Perioperative Neurocognitive Disorder (PND) to capture the range of associated conditions [125].

Evidence suggests modifiable factors like smoking, alcohol consumption, obesity, physical inactivity, and poorly controlled diabetes contribute to cognitive decline [126]. While perioperative interventions may have limited impact, a cancer diagnosis and interactions with healthcare professionals offer opportunities for lifestyle modifications with potential long-term cognitive health benefits.

Screening: Despite international guidelines recommending preoperative cognitive screening, the practice is not widespread [125]. The resultant under recognition may be due to the early, subjective nature of symptoms, lack of awareness, absence of gold-standard screening tools and the time-intensive nature of clinical assessment techniques. The American College of Surgeons and the American Society of Anesthesiologists [127] recommend cognitive screening of all patients over the age of 65 to assess risk of PND. Table 4 contains a non-exhaustive list of candidate screening tools of cognitive function.

Assessment: Screening alone is not diagnostic for MCI or dementia; clinical evaluation by specialists like geriatricians, neuropsychiatrists, or neurologists is necessary. Perioperative cognitive assessment covers orientation, attention, language, memory, executive function, praxis, visuospatial, neurological function, and general impression (slowness, inappropriateness, mood) [132], feeding into cognitive rating scales such as Addenbrooke’s Cognitive Examination, which are sensitive to detection of early dementia [133]. At-risk individuals should be referred to geriatrician or neurology services for comprehensive pre and postoperative care, including addressing modifiable risk factors like inappropriate medication use [134]. Tailored discussions should cover the potential for postoperative delirium, extended hospital stay, and higher chances of discharge to a care facility instead of home [125].

### 2.6. Prehabiltation

Definition: Initially focusing on exercise, prehabilitation has rapidly evolved into a multimodal model that encompasses exercise, nutrition, psychological support, smoking cessation, and alcohol use moderation, underpinned by supported behaviour change, to address the modifiable components of functional capacity and frailty [135].

**Figure 1:**
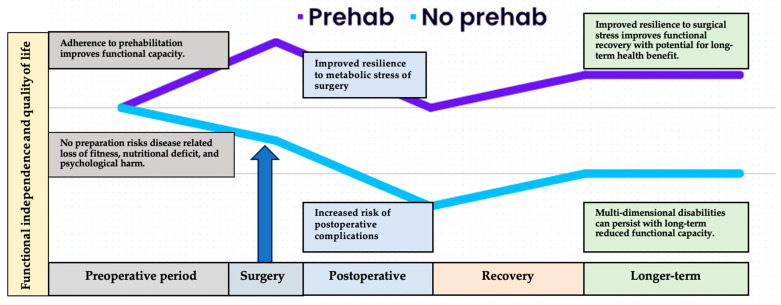
Overview of the differential health trajectories related to adherence to prehabilitation prior to surgery. The cancer care continuum begins at the point of diagnosis and contemplation of surgery. While the health system works through necessary diagnostic and administrative processes, the patient can begin to prepare to face the metabolic stress of surgery. Those who arrive at the operating theatre physically fit, nutritionally replete, and psychologically prepared are likely to suffer less severe response to surgical stress, recover more quickly and more fully, and regain previous or improved functional capacity and quality of life in the longer-term postoperative period.

History: Cancer survivorship, integral to gold-standard, person-centred care, spans from diagnosis to end of life. In 2006, a pivotal report by the Institute of Medicine and the National Research Council emphasized the need for focused attention on the “period following first diagnosis and treatment” [136]. While traditional perioperative rehabilitation reacted to treatment-induced impairment, widely adopted enhanced recovery after surgery (ERAS) programs adopted a proactive stance. These programs, grounded in early postoperative reintroduction of physical activity and nutrition, aimed to prevent surgery-associated morbidity, and facilitate early hospital discharge [137]. Building on this progressive model of care, prehabilitation is a multidisciplinary approach that aims to optimise a person’s physical and emotional resilience in preparation for the upcoming surgical procedure (see Figure 1).

Evidence: Existing research offers promising yet inconclusive evidence of the impact on cancer surgery outcomes. While certain studies suggest a shorter hospital stay (LOS) and a reduced occurrence of postoperative complications [138,139,140], others report non-significant effects on these outcomes [138,141]. Emerging evidence suggests that exercise during neoadjuvant therapy (NAT) not only mitigates associated mitochondrial degradation [142], but may improve treatment tolerance, reduce toxicity, and augment tumour regression [143]. This finding gains biological plausibility from murine models, making it appealing and deserving of substantial investment in well-conducted controlled research trials [144] Additional systematic reviews have reported positive effects on quality of life, functional capacity, and body composition, but express caution due to methodological limitations [145,146,147]. The conflicting evidence has been attributed to heterogeneity of prehabilitation programmes, training principles, patient populations, variations in outcome measures, and inadequate a priori power calculations, leading to calls for standardisation and robust trial design in advance of large-scale clinical effectiveness evaluation [139,148,149,150].

Key principles: Despite acknowledging the need for cautious interpretation of the evidence, calls have grown for widespread adoption of surgical prehabilitation [33,151] and a range of foundation principles have emerged to address the issues associated with preoperative risk.

**Figure 2:**
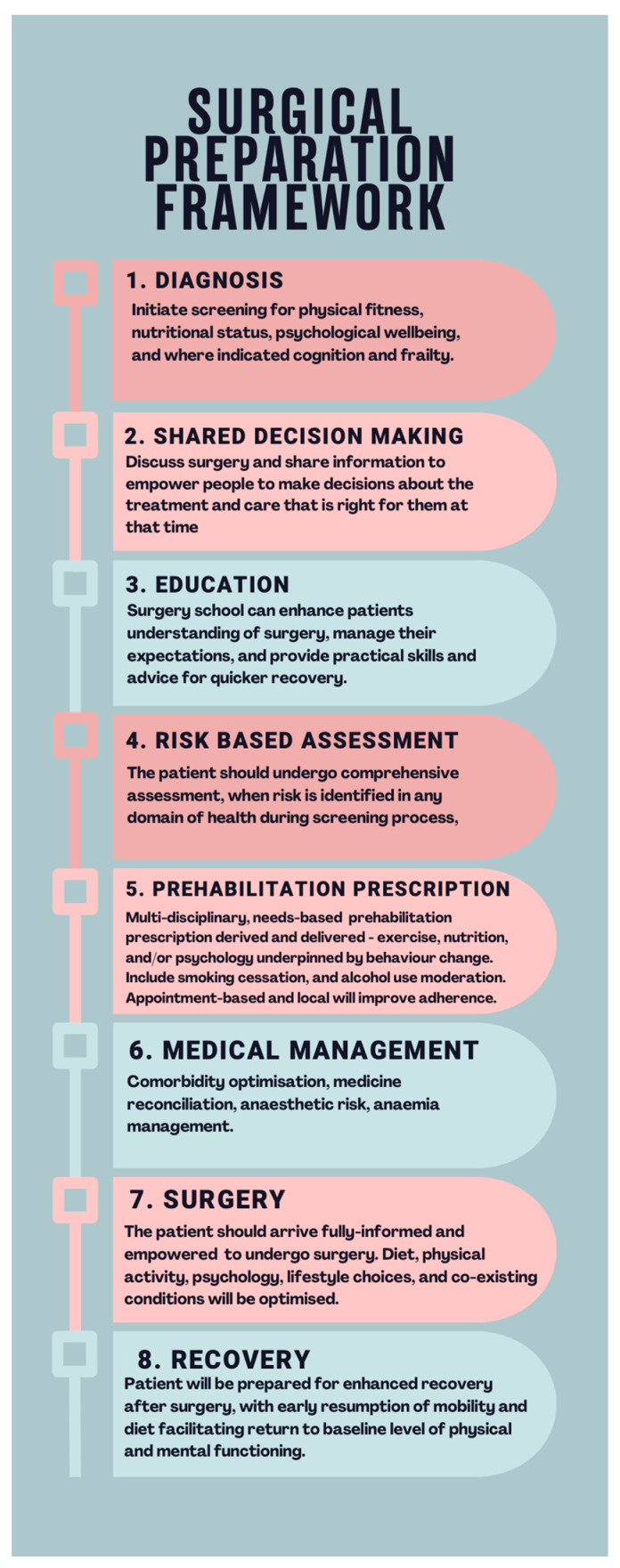
Overview of the proposed framework for optimal preparation prior to surgery. The period between diagnosis and surgery can be repurposed from waiting time to preparation time. This is reliant upon early screening for risk and targeted assessment, informing meaningful shared decision-making, surgical education, and personalised prehabilitation prescription, underpinned by behaviour change, established medical management, and enhanced recovery techniques.

1. Detailed mapping of patient pathways and establishment of efficient clinical framework to support large throughput of numbers in the time limited gap between diagnosis and surgery. Figure 2 illustrates a proposed evidence-based framework for preoperative management that incorporates prehabilitation [135].
2. Multimodal prehabilitation programmes which incorporate the foundational pillars of prehabilitation, physical fitness, nutrition, and psychological support are likely to be the most effective. Patients may require a tailored focus on individual elements based upon screening and assessment of need [42].
3. The multidisciplinary team is required to support multimodal prehabilitation, with specialist assessment and individualised prescription [42]. Complexity of the intervention means clinicians interested in developing prehabilitation services should identify and engage with key stakeholders, including funders, as early as possible in the development process [152,153].
4. Co-designing services with patient representatives to promote a culture of supported self-management rather than being solely directed by healthcare professionals [14,154].
5. Recognition of challenges faced by patients in making lifestyle changes when faced with the effects of cancer diagnosis and upcoming treatment. Appointment-based, local, and supervised facilities can improve adherence to prehabilitation routines [155].
6. Exercise prescriptions should adhere to international guidance, aiming for 150 min of moderate intensity aerobic exercise per week, or 75 min of vigorous intensity aerobic exercise per week. Given the time pressed nature of the preoperative period, high-intensity interval training (HIIT) sessions are a safe, effective, and time-efficient method of improving physical fitness [156]. Patients should also complete two sessions of strengthening exercises per week. Patients with pre-frailty may benefit from additional balance and strength training [157].
7. Improving nutritional status supports increased physical activity and exercise, and may halt or correct cancer cachexia and improve body composition [152,158]. There is little evidence to support universal dietetic counselling; however, signposting to healthy-eating resources is recommended by prehabilitation guidance [33]. Patients identified at intermediate risk through unintended weight loss, moderate weight loss, and/or unfavourable body composition, as well as those increasing their physical activity and exercise, may also benefit from targeted dietetic counselling and/or oral nutritional supplementation [159], supervised by qualified dietetic professionals [158]. This does assume a functioning gastrointestinal tract. Where oral nutrition and supplementation does not meet elevated metabolic demands, enteral supplementation would be preferred over the parenteral route, which should only be delivered under professional prescription in a specialist inpatient setting [158].
8. Psychological prehabilitation is less well studied; however, patients with cancer who have anxiety and depression should receive targeted behavioural techniques such as relaxation, counselling, and emotion management interventions [160]. Patients with pre-existing and/or severe psychopathology should receive specialist psychological or psychiatric therapies [88].
9. Data collection using variables that capture patient experience and healthcare resource utilisation as a key determinant of sustainable funding [157,158].
10. ‘Surgery schools’ provide information on what patients can expect before and after surgery, and instruction in self-management of their preparation for surgery through behavioural change. Schools should deliver accepted guidance on increasing physical activity, nutrition, weight management, smoking cessation, and alcohol consumption in line with government guidelines [161].

## 3. Conclusions and Recommendations

The field of surgical cancer care has made remarkable gains in its mission to save lives. However, for the individuals in our care, the risk of postoperative complication looms large and can cause a significant and negative change to life trajectory for the entire family. We are facing a large, predicted increase in demand for cancer surgery and are further challenged by limited resources. It is imperative therefore that clinicians find scalable, effective, and economically viable methods to improve an individual’s postoperative outcome, improve the health of the population, and reduce per capita costs. The next challenge for healthcare providers is to embrace the opportunity provided by utilising the time between diagnosis and surgery to prepare the patient for the upcoming psychological and physiological stress. Comprehensive care should be offered within the entire cancer care continuum, from diagnosis to complete recovery.

This comprehensive review underscores the intricate relationship between multiple components of functional capacity and cancer care outcomes, particularly in the context of surgical interventions. It advocates for a refined risk evaluation approach that encompasses universal screening, followed by targeted assessment for patients at heightened risk. We have further highlighted the evidence-based foundational principles of prehabilitation. The detailed assessment can be used to guide individualised prehabilitation prescriptions to optimise patient condition prior to surgery and contribute to improved patient outcomes. Moreover, truly informed consent and meaningful shared decision-making discussions must be predicated upon accurate quantification of surgical risk.

Functional capacity is a widely recognised component of patient resilience, facilitating a proportionate response to the stress associated with cancer diagnosis and subsequent surgery. Traditional assessment approaches are empirically proven inaccurate predictors of risk [27]. This may in part be due to a narrow definition of functional capacity, overly reliant on assessment of purely physical ability. Evidence suggests that a true assessment of perioperative risk should encompass a broader definition, incorporating physical fitness, nutritional status, psychological health, and, where indicated, frailty and cognitive health.

Largely developed in the perioperative context, prehabilitation is now recognised as a key element of the wider cancer care continuum, empowering individuals to increase personal resilience in the face of challenges posed by preventative, restorative, supportive, and palliative phases.

To advance the field of prehabilitation research, several key recommendations emerge. Firstly, interventions should be tailored to individual patient needs and preferences and tested for effectiveness and impact. Secondly, the incorporation of implementation science methodologies is essential to bridge the gap between research and clinical practice, facilitating the integration of prehabilitation into routine patient care. Additionally, conducting health economics analyses can provide crucial insights into the cost-effectiveness and resource allocation aspects of prehabilitation programs, which can be instrumental in decision-making processes. Thirdly, a commitment to rigorous trial design, particularly randomised controlled trials, is fundamental to establishing the causal relationships and efficacy of prehabilitation interventions. Furthermore, the establishment of core outcome datasets will harmonise research efforts, enabling meaningful comparisons across studies and facilitate evidence synthesis.

Cancer surgery is a risky treatment, associated with high rates of mortality and morbidity, healthcare cost burden, and deleterious effects on quality of life for survivors. Addressing the associated risk has the potential to improve lives and save money. Addressing this risk in such a safety critical clinical environment provides both challenge and opportunity.

## Figures and Tables

**Table 1:** Candidate physical fitness screening tools. These proposed tools are widely used and validated in surgical populations, but the list should be regarded as a guide rather than definitive. Perioperative teams should choose tools most appropriate for their clinical context, culture, and populations.

Screening Tool	Summary
Duke Activity Status index (DASI)	Self-reported measure of fitness and function, correlates to CPET variables [27] and predictive of postoperative outcome [36].
International Physical Activity Questionnaire (IPAQ)	A 27-item self-reported measure, the IPAQ offers a comprehensive evaluation of various aspects of physical activity, including intensity, duration, and frequency.
Godin Shephard Leisure Time Physical Activity Questionnaire (GSLTPAQ)	Short, 4-question measure, commonly used in oncology research, that categorises individuals according to level of physical activity against published guidelines.

**Table 2:** Selection of nutrition screening tools with included components.

Screening Tool	Included Components						
	Body Weight	Body Mass Index	Unintended Weight Loss	Dietary Intake	Symptoms Affecting Intake	Function	Biomarkers
Patient-Generated Subjective Global Assessment (PG-SGA) [56]	X	X	X	X	X	X	
Malnutrition Screening Tool (MST) [58]			X	X			
Royal Marsden Nutrition Screening Tool (RMNST) [59]			X	X	X		
Perioperative nutrition screen (PONS) [60]	X	X	X	X			X
Malnutrition Universal Screening Tool (MUST) [61]	X	X					

**Table 3:** Frailty screening tools.

Screening Tool	Description
Clinical Frailty Scale (CFS) [112]	Simple pictorial scale providing nine pictures and written descriptions ranging from 1 ‘very fit’ to 9 ‘terminally ill’.
Edmonton frailty Scale (EFS) [113]	Covers nine components of health: cognition, general health, self-reported health, functional independence, social support, polypharmacy, mood, continence, and functional performance. Scored out of 17 with patients considered ‘not frail’ (0–5), ‘apparently vulnerable’ (6–7), ‘mildly frail’ (8–9), ‘moderately frail’ (10–11), or ‘severely frail’ (12–17)
FRAIL Index [114]	Screens for presence of fatigue, resistance, ambulation, illness, and loss of weight. Presence of 3 or more items is regarded as marker of frailty, 1–2 items is ‘pre-frailty’ and 0 items is ‘robust’.

**Table 4:** Cognitive function screening tools.

Screening Tool	Description
Montreal Cognitive Assessment (MoCA) [128]	A brief screening tool (10 min) for cognitive function. High sensitivity and specificity for detecting MCI.
Mini-mental state examination [129]	A brief examination of cognitive function domains; orientation to time and place, registration, attention and calculation, recall, language, repetition, and complex commands.
Mini-Cog [130]	The Mini-Cog is brief (3 min), simple test of recall and a scored clock-drawing test. It can be used after brief training and results are evaluated by a health provider to determine the need for a full-diagnostic assessment.
Confusion Assessment Method [131]	Sensitive and specific test performed postoperatively, the CAM assesses fluctuating cognition, consciousness level, inattention, and disordered thinking. Includes an intensive care-specific version.
